# Features of the normal choriocapillaris with OCT-angiography: Density estimation and textural properties

**DOI:** 10.1371/journal.pone.0185256

**Published:** 2017-10-11

**Authors:** Giovanni Montesano, Davide Allegrini, Leonardo Colombo, Luca M. Rossetti, Alfredo Pece

**Affiliations:** 1 University of Milan, School of Ophthalmology, Milan, Italy; 2 ASST Santi Paolo e Carlo–Eye Clinic, Milan, Italy; 3 City, University of London, Optometry and Visual Sciences, London, United Kingdom; 4 Eye clinic, Humanitas Gavazzeni, Humanitas University, Milan, Italy; 5 Eye Clinic, Melegnano Hospital, Vizzolo Predabissi, Italy; Medizinische Universitat Graz, AUSTRIA

## Abstract

The main objective of our work is to perform an in depth analysis of the structural features of normal choriocapillaris imaged with OCT Angiography. Specifically, we provide an optimal radius for a circular Region of Interest (ROI) to obtain a stable estimate of the subfoveal choriocapillaris density and characterize its textural properties using Markov Random Fields. On each binarized image of the choriocapillaris OCT Angiography we performed simulated measurements of the subfoveal choriocapillaris densities with circular Regions of Interest (ROIs) of different radii and with small random displacements from the center of the Foveal Avascular Zone (FAZ). We then calculated the variability of the density measure with different ROI radii. We then characterized the textural features of choriocapillaris binary images by estimating the parameters of an Ising model. For each image we calculated the Optimal Radius (OR) as the minimum ROI radius required to obtain a standard deviation in the simulation below 0.01. The density measured with the individual OR was 0.52 ± 0.07 (mean ± STD). Similar density values (0.51 ± 0.07) were obtained using a fixed ROI radius of 450 μm. The Ising model yielded two parameter estimates (β = 0.34 ± 0.03; γ = 0.003 ± 0.012; mean ± STD), characterizing pixel clustering and white pixel density respectively. Using the estimated parameters to synthetize new random textures via simulation we obtained a good reproduction of the original choriocapillaris structural features and density. In conclusion, we developed an extensive characterization of the normal subfoveal choriocapillaris that might be used for flow analysis and applied to the investigation pathological alterations.

## Introduction

The choroid is a multifunctional vascular structure, whose main functions range from providing nourishment for the external layers of the retina to thermal regulation [1]. This vascular structure is usually divided into 5 layers (from the closest to the retina): the Bruch’s membrane, the choriocapillaris, the Haller’s layer, the Sattler’s layer and the suprachoroid [1]. Recent development of Optical Coherence Tomography Angiography (OCT-A) techniques has allowed a more extensive imaging of the living eye vasculature. These imaging techniques rely on the light scattering produced by moving blood cells during OCT acquisition to obtain enhanced contrast of perfused retinal and choroidal vessels [2]. Differently from the retinal vascular system, the choroidal vasculature is not fully resolved by OCT-A: structures below the choriocapillaris are masked by the highly scattering overlaying tissue, although the Sattler’s layer can be partially visualized [2]. Thus, the choriocapillaris is, at present, the most clearly investigable choroidal tissue with OCT-A [3]. It’s implication in several retinal diseases, both hereditary and age related, is raising interest for this peculiar vascular structure and its features in OCT-A [4].

Detailed visualization of the choriocapillaris has long been limited by conventional dye based methods (Fluoresceing Angiography, FA, and Indocyanine Green Angiography, ICG-A) due to scattering of fluorescent signal from retinal tissues and leakage from choroidal and retinal vessels [4]. Choriocapillaris is an intricate anastomosed network of fenestrated capillary vessels of 20–40 μm in diameter, with an overall 10 μm thickness at the subfoveal location and 7 μm at the periphery [2]. In OCT-A imaging, it appears as a mesh-like homogeneous tissue and single vessels are usually not discernible [2].

Although some attempts have been made to provide quantitative description of this complex network [4, 5], a careful characterization of its features in normal subjects is still lacking. We mostly focused on the subfoveal region for two main reasons: on one hand, it is the region most likely related to the visual function; on the other hand, it is a homogeneous vascular network less affected by projection artifacts from superficial retinal vessels, since most of its area is covered by the Foveal Avascular Zone (FAZ). We decided to focus on two main aspects: 1) the accurate estimation of the subfoveal choriocapillaris vascular density and 2) the characterization of the textural properties of the subfoveal choriocapillaris.

For the first aim, we used a Monte-Carlo approach to obtain optimal measures of the vascular density on binarized images of the choriocapillaris OCT-A. Monte-Carlo methods are a class of simulation strategies able to provide estimation of parameters when complex or unidentified distributions are involved and have been proven extremely useful in biomedical problems and visual neuroscience [6–8]. In our case, using circular Region of Interest (ROI) of different radii with small random displacement of their center, we calculated the variance of the density measurements and defined an optimally small circular area to obtain stable measurements. For the second aim, we estimated the textural properties of the binarized choriocapillaris estimating the parameters of an Ising Model and simulated realization from the model to check its ability of reproducing the original texture. The Ising model is a special case of binary Markov Random Field (MRF) that deals with binary processes on a regular lattice and is widely recognized, along with other random fields, as a precious tool for texture analysis, texture synthesis and image segmentation in biomedical and retinal imaging [9–12] and here we employed its precious abilities of modeling complex spatial patterns of the irregular textures of the choriocapillaris layer.

## Methods

We retrospectively analyzed 32 healthy eyes from 25 patients from an Eye clinic in Milan (Retina 3000 Foundation Eye Clinic). The Retina 3000 Foundation does not require the evaluation from the IRB for retrospective anonymous analysis on imaging data acquired during normal clinical practice. However, all participant provided explicit informed consent for any future treatment of the data for research purposes in anonymous form at the moment of examination. Our research adhered to the tenets of the Declaration of Helsinki. Informed consent for the treatment of the data was acquired from each patient. All data were anonymized prior to analysis. OCT data were acquired with RTVue XR Avanti SD-OCT (OptoVue) with a 3 mm x 3 mm scanning protocol. OCT-A data were obtained with the SSADA (Split Spectrum Amplitude Decorrelation Analysis) algorithm and retinal layers were segmented according to the automated segmentation provided by the built in software. Images of the superficial vascular layer and the choriocapillaris were exported as RAW data and read using a MATLAB (MATLAB, The MathWorks Inc., Natick, MA, 2000) routine. Only high quality images were selected for this analysis, being especially careful to avoid images with motion or blink artifacts.

### Image processing

**Fig 1:**
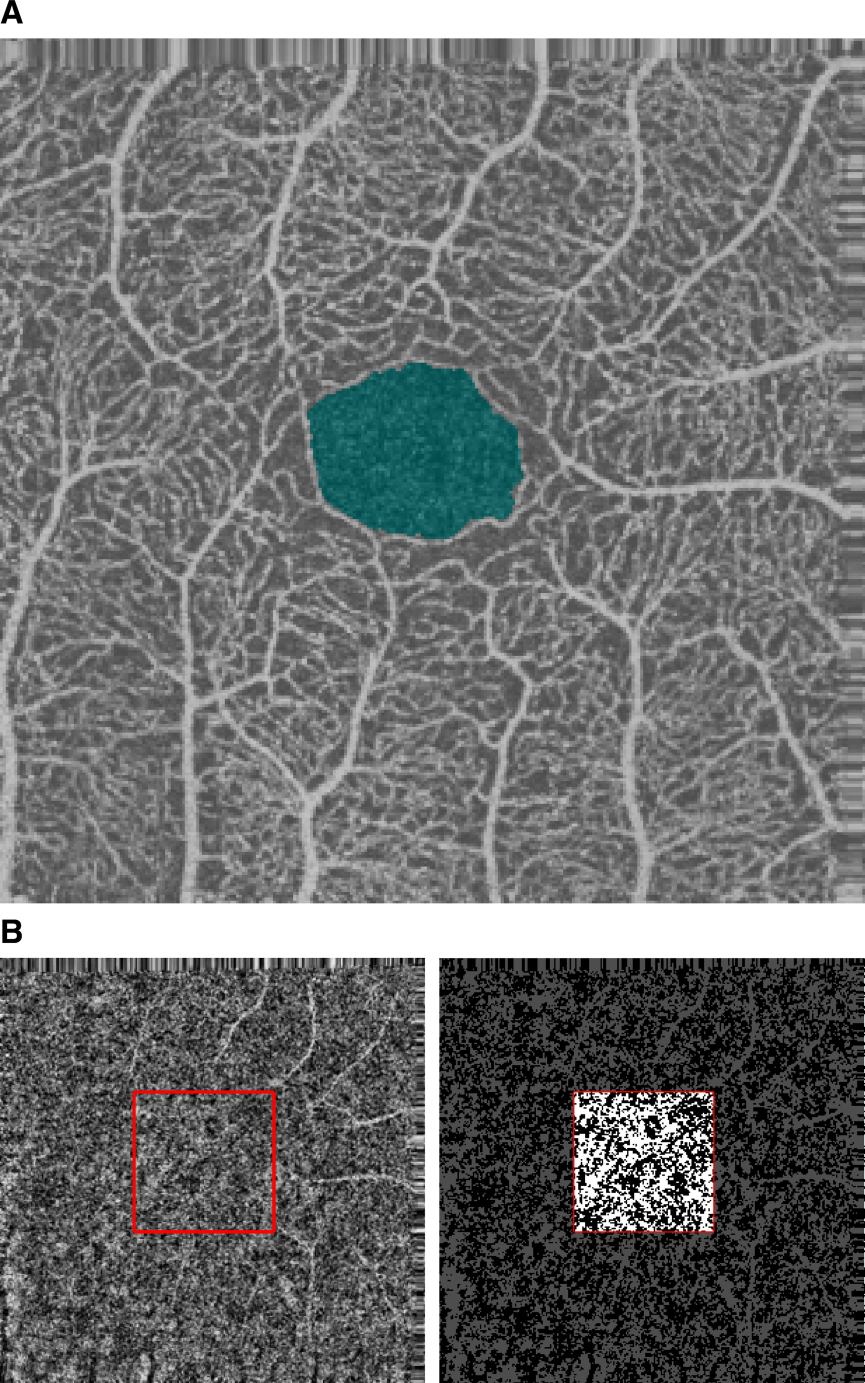
FAZ identification and choriocapillaris binarization. Fig 1.A shows an example of normal superficial vascular layer of the retina. The FAZ is highlighted in blue. Fig 1.B shows the original choriocapillaris of the same subject (on the left) with the central 100 x 100 pixel area considered for the estimation of the parameters of the Ising model highlighted by a red square; the same area highlighted on the thresholded version of the same image to create a binary image for density calculation. Notice the projection artifacts from superficial vessels on the right side of the image and how they are excluded by considering the central part of the image.

The FAZ was automatically detected using a custom made MATLAB routine (see Fig 1A). Briefly, we thresholded the superficial OCT-A image and calculated the distance map on the binarized image. Edges of the FAZ were detected as the pixels with a value on the distance map less than 3 to exclude any artifactual bridges between contiguous avascular zones occurring during thresholding (Fig 1A). For few (2) cases with very small FAZs, detection was not accurate and had to be manually corrected.

Choriocapillaris OCT-A image was transformed in a binary image for density analysis using a custom made MATLAB routine. Briefly, we equalized the image using the adaptive histogram equalization algorithm provided by MATLAB with the default setup and then we thresholded the equalized image at a fixed threshold of 0.3 (Fig 1B). For all the analyses we used images resulting from the projection artifact removal algorithm of the RTVue XR Avanti software.

### Simulation for density measure stability

Throughout the paper, for the choriocapillaris, we define density (calculated on the binary thresholded image) as the number of white pixels over the total number of pixels in the region considered. This number is adimensional. To assess the minimum extension of the circular Region Of Interest (ROI) required to obtain stable measurements of the subfoveal choriocapillaris density we used a simulation approach: firstly, we used the centroid of the FAZ detected on the superficial layer as the barycenter of the simulation; we then performed, for each image, 10000 random measurements of the choriocapillaris density within circular areas; for each measurement, we extracted a random radius length from a uniform discrete distribution on the interval [10, 1000] μm with 10 μm spacing (corresponding to the minimum spatial resolution, i.e. one pixel increase); then, we independently sampled a random displacement from the barycenter from a bivariate isotropic normal distribution with zero mean (*μ = 0*) and *σ* = 10 pixel (100 μm) to simulate small errors in the manual placement of the measuring circular area that could occur during common clinical practice. We combined these two random extractions to generate random ROIs with random displacement from the barycenter and random radius length; in each random ROI we measured the choriocapillaris density. We used these measurements to calculate the mean and standard deviation (STD) of the measured density at each radius length. We used a cut-off of 0.01 on the standard deviation to calculate the optimal radius length, defined as the minimum radius length that allowed a STD of the measured density <0.01.

All calculations for this section were performed using custom MATLAB routines.

### Ising model for texture characterization

We used the Ising model to characterize the texture properties of the binary images of the choriocapillaris. The Ising model is a special case of binary Markov Random Field (MRF) that deals with binary processes on a regular lattice, originally proposed by Ernst Ising to model spin interaction in ferromagnetic materials. Here, for ease of computation, all white pixels were equal to 1 and all black pixels equal to -1. Let *I* be the set pixels in the image. A binary random field is then a collection of binary random variables {*X*_*i*_: *i* ∈ *I*}. We denote the neighbors of *i* by *δi*. A random field is called a Markov random field (MRF) if the conditional distribution of any pixel (*x*_*i*_) given the values of all other pixels (*x* − *i*) only depends on the values of its neighbors,

$$P(x_{i}|\;x-i)=P(x_{i}|\;\delta i)$$ (1)

We chose a 4-element neighborhood system, including the closest pixels on the vertical and horizontal axis. Then, the probability of a certain configuration of black and white pixels depends on the neighboring system according to

$$P(X)=\frac{1}{Z}\exp\left(-U(X)\right)$$ (2)

where *U* is the potential energy of the configuration *X* (the collection of all the outcomes of the random variables in a particular realization of the random field). *U* is defined as [13]

$$U(X)=-\beta \sum_{i\in I,\;j\in \delta i}x_{i}x_{j}$$ (3)

where the summation is taken over all edges; *Z* is the partition function, or the summation of the exponential of potential energy over the set of all possible configurations (Ω) of black and white pixels in the chosen image

$$Z=\sum_{X\;\epsilon \;\Omega }exp(-U(X))$$ (4)

*β* defines the ‘attractive’ parameter, regulating how pixels with similar values tend to cluster together, with higher values associated with higher clustering. Since, for this kind of models, it is not possible to calculate *U*(*X*) for all possible configurations (and thus the constant *Z*), it is impossible to obtain Maximum Likelihood Estimates (MLEs) of the parameters (in the following formula generically denoted as *ϑ*). Therefore we estimated the parameters using a Maximum Pseudo Likelihood Estimate (MPLE) introduced by Besag [14] which uses log-Pseudo Likelihood (*logPL*) goodness of fit indicator based only on local indicators

$$logPL\;(\vartheta )=\sum_{i}ln({P_{X_{i}|X_{\delta i}}^{\vartheta }}_{}\;(x_{i}|x_{\delta i}))$$ (5)

The model defined above only accounts for clustering of the pixels and, for each configuration, its inverse is equally probable. To ensure reproduction of the correct density of the true choriocapillaris we introduced an additional parameter that modeled the preferential prevalence of white or black pixels.

So the full Hamiltonian (or total potential energy, *H*) of the system is defined as

$$H(x)=-\beta \sum_{i\in I,\;j\in \delta i}x_{i}x_{j}-\gamma \sum_{i\in I}x_{i}$$ (6)

where a positive (negative) value of the second parameter *γ* makes the presence of white (black) pixels more likely. The MPLEs of the two parameters where obtained for each image using a numeric maximization of the following *logPL* over the parameter space.

$$logPL\;(\beta ,\gamma )=\sum_{i}\log\left(\frac{\exp\left(\beta \;\sum_{j\in \delta i}x_{i}\;x_{j}+\gamma \;x_{i}\right)}{2\;\cosh\left(\gamma +\beta \;\sum_{j\in \delta i}\;x_{j}\right)}\right)$$ (7)

We then used a classic Metropolis-Hastings Markov Chain Monte Carlo (MH-MCMC)[15] to generate random realizations of the Ising model with the estimated parameters to compare the results from the model with the original images.

All calculations for this section were performed using custom MATLAB codes.

## Statistical analysis of the results

A variety of linear models, have been used throughout the paper. Within the frame of linear models, we used random effects deal with correlated observations (especially measurements from the two eyes from the same subject, or repeated measures from the same eye).

All statistical analyses were performed in the R scripting environment.

## Results

### Study population

We retrospectively reviewed images from patients that referred to our clinic from June 2015 to July 2016. We selected patients with 10/10 Best Corrected Visual Acuity (BCVA) in the eye considered for the analysis and no evident alterations at the OCT scanning in the same eye. Other clinical parameters, such as axial length and precise refractive correction, were not available for many patients and thus not considered. In spite of this, it has been shown that, differently from retinal vessels, axial length has no significant effect on choriocapillaris density[5]. Although this parameter could be safely disregarded, we did not select eyes showing evident staphyloma or important alterations in the shape of the posterior pole. Based of these criteria, we selected images of 32 normal eyes out of 25 subjects. Selected subjects had a mean age 49.8 (STD ± 16.4; range 16–86) at the time of the OCT scanning.

### Choriocapillaris density

As explained in the Methods section, we used a simulation approach to calculate the stability of the choriocapillaris density measure with different ROI areas when the position of circular ROIs used for the calculation was perturbed by random displacement from the center of FAZ. Such random displacement was drawn from an isotropic bivariate normal distribution with zero mean and *σ* = 10 pixels; the idea was to obtain an estimate of the minimum area of the measuring ROI required for the measure not to be highly dependent on small variations in ROI’s center position. The importance of detecting the minimum area required relies in the fact that we were particularly interested in the estimation of the subfoveal choriocapillaris density, as the region with less projection artifacts and more related with possible alterations in the visual function.

**Fig 2:**
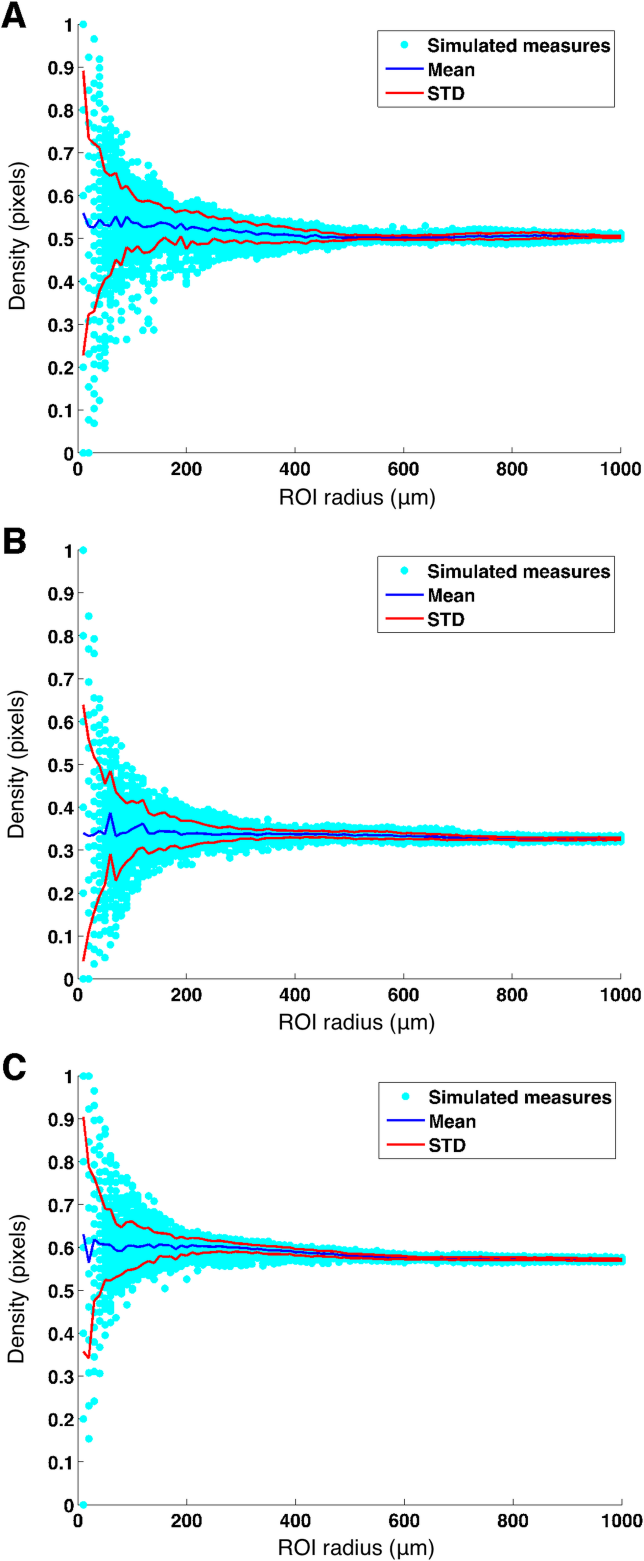
Choriocapillaris density estimation at increasing ROI radius. Fig **2**.A-C shows density estimates of three exemplar choriocapillaris images. Scattered points indicate the random density measures taken on the binarized image of the choriocapillaris with circular ROIs with different radii randomly extracted from a uniform distribution interval [10, 1000] μm with 10 μm spacing and random displacement from the FAZ center (independently sampled from a bivariate isotropic normal distribution with zero mean and σ = 10 pixel (100 μm) to simulate small errors in the manual placement of the measuring circular area). Mean and Mean ± STD were calculated at each radius length an overlaid on the scattered points (blue lines and red lines respectively). Notice how the variability of the measure decreases between 400 and 600 μm radius length.

Such calculation was performed on each of the full 3 x 3 mm (304 x 304 pixel) decorrelation images from the SSADA algorithm, after equalization and thresholding (as described in the Methods section). All calculations have been performed by displacing the circular ROI area on the original images, and no synthetic images were generated. Thus, the simulation was used to randomly displace the circular ROI around the foveal region of each original image several times while increasing the radius of the ROI. At each displacement the density was calculated. Results of the simulations for three representative cases are reported in Fig 2A–2C. Here, for three images with different choriocapillaris density, we plotted the 10000 measured densities from the simulation against the radius (in μm) of the measuring areas. Overlaid on the scatter plot, the mean density value at each radius length (blue line) and the corresponding mean ± STD (red lines) values.

It is evident how the variability of the density measure starts very high with small measuring areas and drastically decreases between 400 and 600 μm. For each image, we calculated the minimum radius length needed to reach below a maximum STD value (arbitrarily chosen to be < 0.01) in the simulations and denoted it as the Optimal Radius (OR). Here, the term Optimal is not related to any particular application. Instead, it refers to the smallest radius that yielded an acceptable variance of the measure for small displacements, in an attempt to reduce the intrasubject variability while including the least possible amount of extrafoveal tissue. The mean value for OR was 420.62 ± 69.9 μm (mean ± STD), ranging from 290 to 620. The choriocapillaris density measured with the individual OR was 0.519 ± 0.07 (mean ± STD). Since we wanted to set a standard usable radius for clinical practice, we rounded the mean value to 450 μm (45 pixels) and used it as a safe Standard for Density Measurement (SDM). The error of this simplification was calculated by computing the difference in the estimated density between the OR area (denoted as the Optimal Density) and the SDM area (denoted as the Standard Density), both centered on the center of the FAZ, which was 0.0014 ± 0.013 (p = 0.62). The choriocapillaris density measured with the SDM was 0.517 ± 0.07 (Mean ± STD).

Both the Optimal Density and the Standard Density were weakly (but significantly, p = 0.023 and p = 0.048 respectively) negatively correlated with the Age of the subjects, but no correlation could be found with the OR (see Table 1 and Table 2 for numerical values and correlation coefficients).

**Table 1:** Descriptive statistics of important variables.

	*Mean*	*STD*
Age	49.8	± 16.4
OR (μm)	420.62	± 69.9
OD	0.519	± 0.074
SD	0.517	± 0.074
β-parameter	0.34	± 0.03
γ-parameter	0.003	± 0.012

Table 1 reports mean and standard deviation of different variables mentioned within the paper. STD = Standard Deviation; OR = Optimal Radius; OD = Optimal Density; SD = Standard Density

**Table 2:** Correlation of different quantities tested.

	*Dependent variable*:						
	SD		OD		β-parameter	Density of Square Image	
						Univariate	Multivariate
Age	-0.002**		-0.002**				
	(0.001)		(0.001)				
OR		-0.00003		-0.0002*			
		(0.0001)		(0.0001)			
γ-parameter					-1.045***	5.337***	5.507***
					(0.379)	(0.468)	(0.504)
β-parameter							0.191
							(0.205)
Intercept	0.606***	0.523***	0.619***	0.590***	0.345***	0.487***	0.421***
	(0.049)	(0.043)	(0.047)	(0.045)	(0.005)	(0.006)	(0.071)

Note

*p<0.1

**p<0.05

***p<0.01

Table 2 reports the regression coefficients (along with the respective standard errors in brackets) describing meaningful relationships of important variables with each other. In the last two columns, the Density of Square Image refers to the density of the square area on which the Ising model parameters were estimated (Univariate refers to the correlation of such density with the γ-parameter alone and Multivariate with the β and γ-parameter simultaneously). STD = Standard Deviation; OR = Optimal Radius; OD = Optimal Density; SD = Standard Density

To explore any influence of media opacities and other characteristics affecting the image quality, we analyzed the relationship between the calculated densities and the Signal Strength Index (SSI) provided by the device. Although all subjects were healthy with 10/10 BCVA we found a wide range of SSI values (70.19 ± 7.99, Mean ± STD; Range = 55–88). The SSI was strongly correlated with the age of the subject (-0.27 ± 0.08 per year, Estimate ± Standard Error, p = 0.0025). The Optimal Density was weakly correlated with the SSI (0.004 ± 0.001, p = 0.028) while the Standard Density was not (0.0026 ± 0.001, p = 0.12).

**Fig 3:**
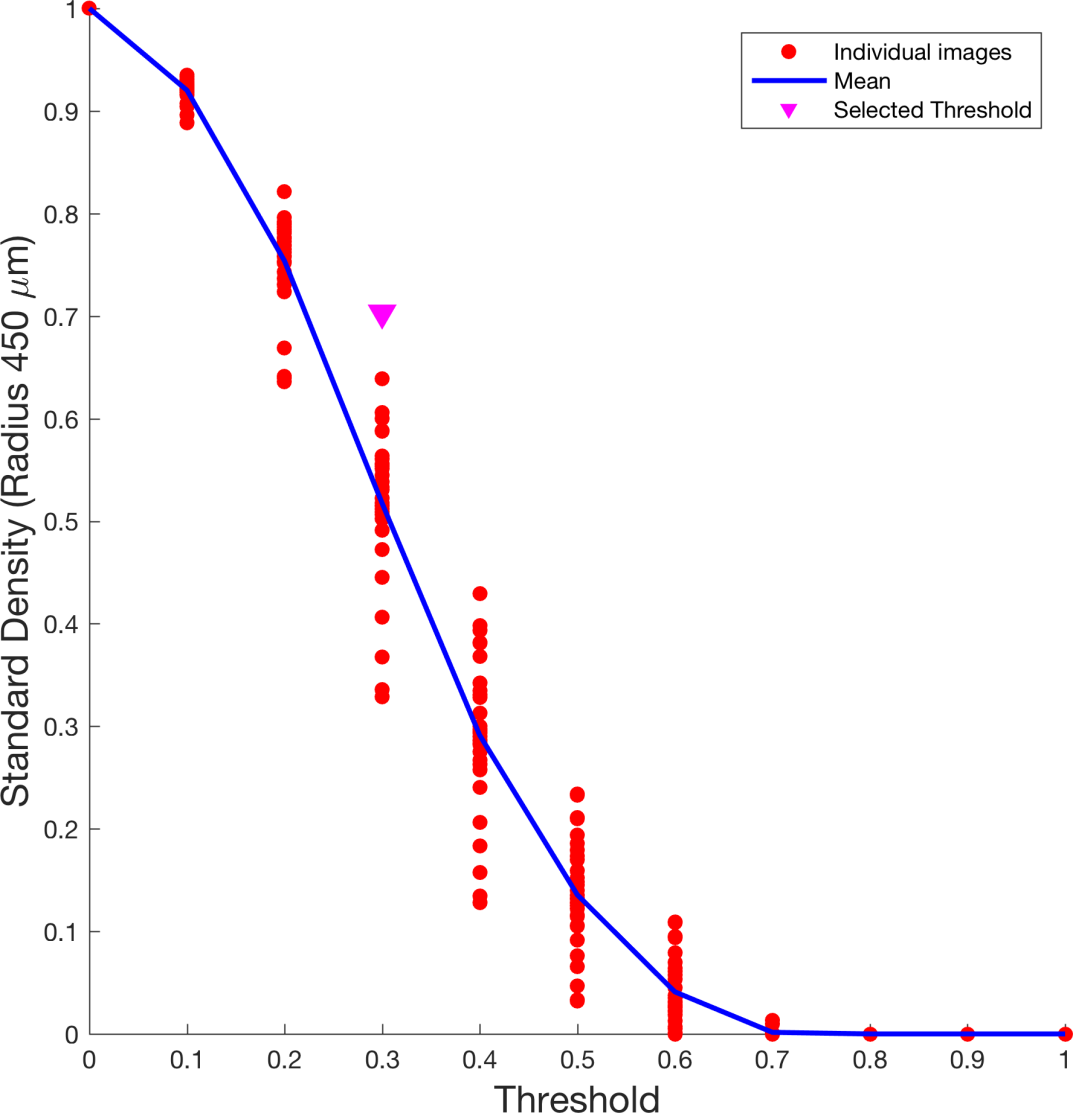
Effect of threshold selection on Standard Density estimates. The figure shows how density estimates in a circular area with a radius of 450 μm (Standard Density) change when using different threshold values to obtain binary images from decorrelation agiograms of the choriocapillaris. The threshold has been increased at 0.1 steps and applied to all images each time to calculate the density. The blue line represents the mean density value at each threshold value, the red dots represent the value calculated for each image and the pink arrow point highlights the threshold value used in this study. Note how the selected value is centered on the sigmoid shaped curve of mean densities, allowing a wide range of possible values.

To explore the effects of threshold selection on the Standard Density measure we calculated density values while changing the threshold used to binarize the choriocapillaris image. The results are depicted in Fig 3. Threshold was increased by 0.1 at each step, from 0 to 1. As expected, the mean Standard Density value decreased at increasing threshold values in a sigmoid fashion. Fig 3 shows how the selected threshold (0.3) lies on the steepest part of the sigmoid curve, determined as the point of maximum gradient in the curve.

### Texture properties of the choriocapillaris OCT-A

When reviewing the binarized images of the choriocapillaris, we realized that each image, although presenting a scattered appearance in the distribution of the white pixels, had some characteristic spatial features. Since the OCT-A scans of the choriocapillaris, in contrast with the superficial vascular layers, do not have any readily recognizable structure, we decided to use Markov Random Field models (see Methods) to characterize the texture properties of the binarized images. MRFs have many different applications in physics, statistics and, more recently, in image analysis, since they are able to build a model for characterizing a texture and eventually generate synthetic random samples of a new texture with similar characteristics. When dealing with binary images, one simple choice is to work with a specific subtype of MRFs, the Ising model. First introduced to model the spin orientation in a ferromagnetic material, it has been adapted to describe various kinds of phenomena with a binary output.

**Fig 4:**
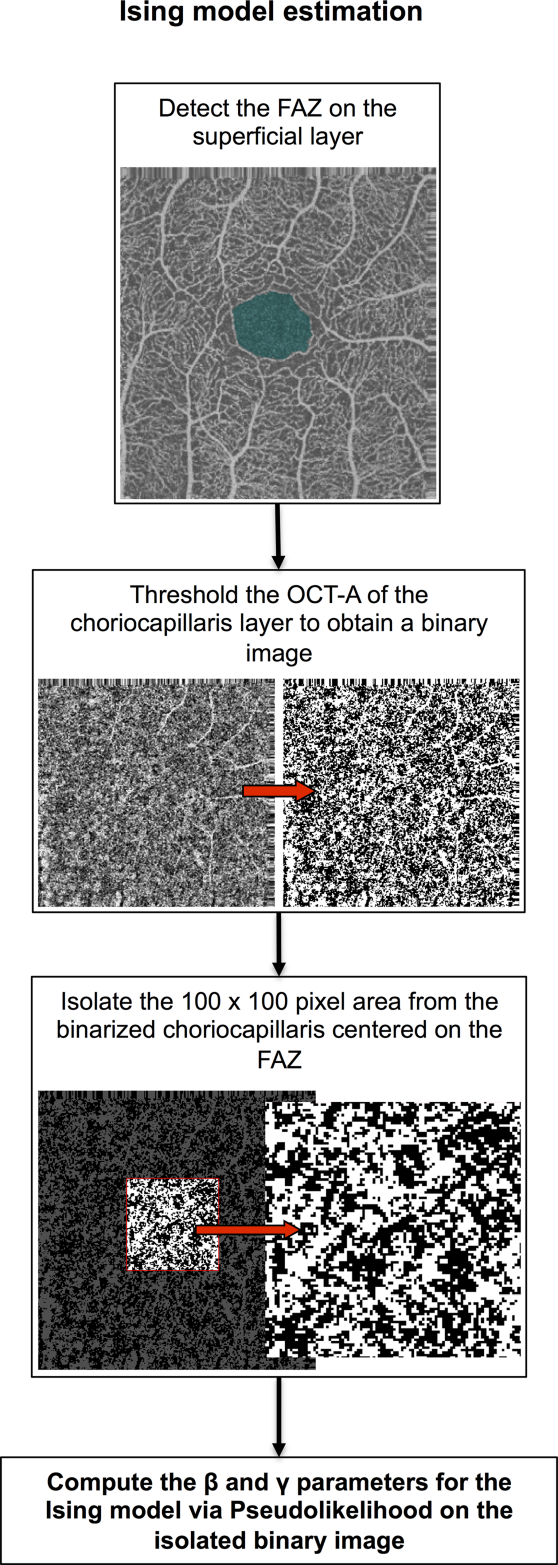
Block diagram of the image processing steps for the Ising model. The block diagram shows the preprocessing steps used to obtain the final binary image used to calculate the parameters of the Ising model. In the first step we identified the FAZ on the superficial layer; then we thresholded the choriocapillaris image as described in the Method section (fixed threshold after adaptive histogram equalization); finally, we selected the central 100 x 100 pixel area centered on the FAZ from the thresholded and used that to estimate the β and γ parameters.

To obtain stable results, we concentrated on the central 100 x 100 pixel subfoveal area centered on FAZ. In the previous section we showed how a 90 pixel was the minimally sufficient diameter of a circular area for a stable estimation of choriocapillaris density. A slightly larger squared area was chosen for the evaluation of texture properties as the one providing stable texture synthesis (see below) from the estimated parameters of the Ising model upon several trials. In none of the chosen 100 pixel squared areas we could detect any significant projection artifact from the superficial layers (see Fig 1 and the block diagram in Fig 4).

**Fig 5:**
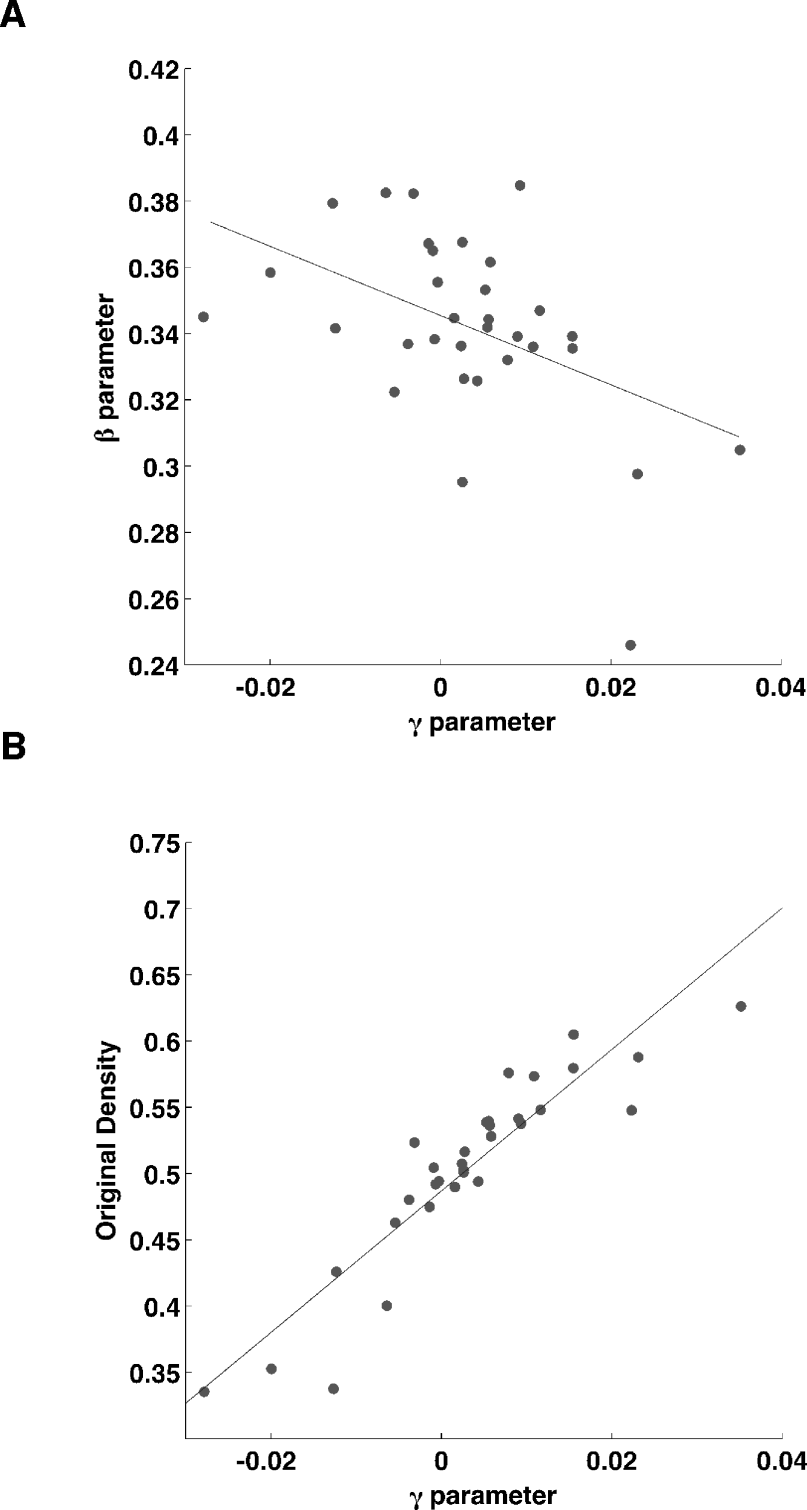
Correlation of the Ising model parameters. Fig 5-A shows a plot of the negative correlation of the two parameters of the Ising model (p<0.01). Fig 5-B shows a plot of the choriocapillaris density of the γ parameter of the Ising model (p<0.01).

In our case, as explained in the Methods section, we used a 4-element neighborhood and estimated two parameters from each image, *β* and *γ*. The first parameter, *β*, can be thought of as a “clustering” parameter, indicating how nearby pixels interact: with higher values, nearby pixels will be more likely to be similar, while, when approaching zero, pixels will be spatially independent. The second parameter, *γ*, represents the preference of the model to favor white pixels (if positive) or black pixels (if negative): thus, this second parameter is mostly related to the overall density of white pixels (see Fig 5B and Table 2). The parameters were estimated via Pseudolikelihood. The procedure for obtaining the Ising estimates is summarized in the block diagram in Fig 4.

The mean value of the estimated *β* parameters was 0.34 ± 0.03 (Mean ± STD) with a coefficient of variation (CV) of 0.08, while the mean value of the estimated *γ* parameters was 0.003 ± 0.012 (Mean ± STD) with a CV of 3.77. The two parameters showed a significant correlation with each other (p<0.01, *β* regressed on *γ*) and the *γ* parameter showed a strong positive correlation with the overall density measured on the considered square area (R^2^ = 0.82, p<0.01, see Fig 5B and Table 2). The two parameters were also used as predictors for the density in a multivariate model (see Table 2); although these two predictors were significantly correlated, the Variance Inflation Factor for the multivariate model was low (1.15); in this case, the *γ* parameter was the only significant predictor (p<0.01).

**Fig 6:**
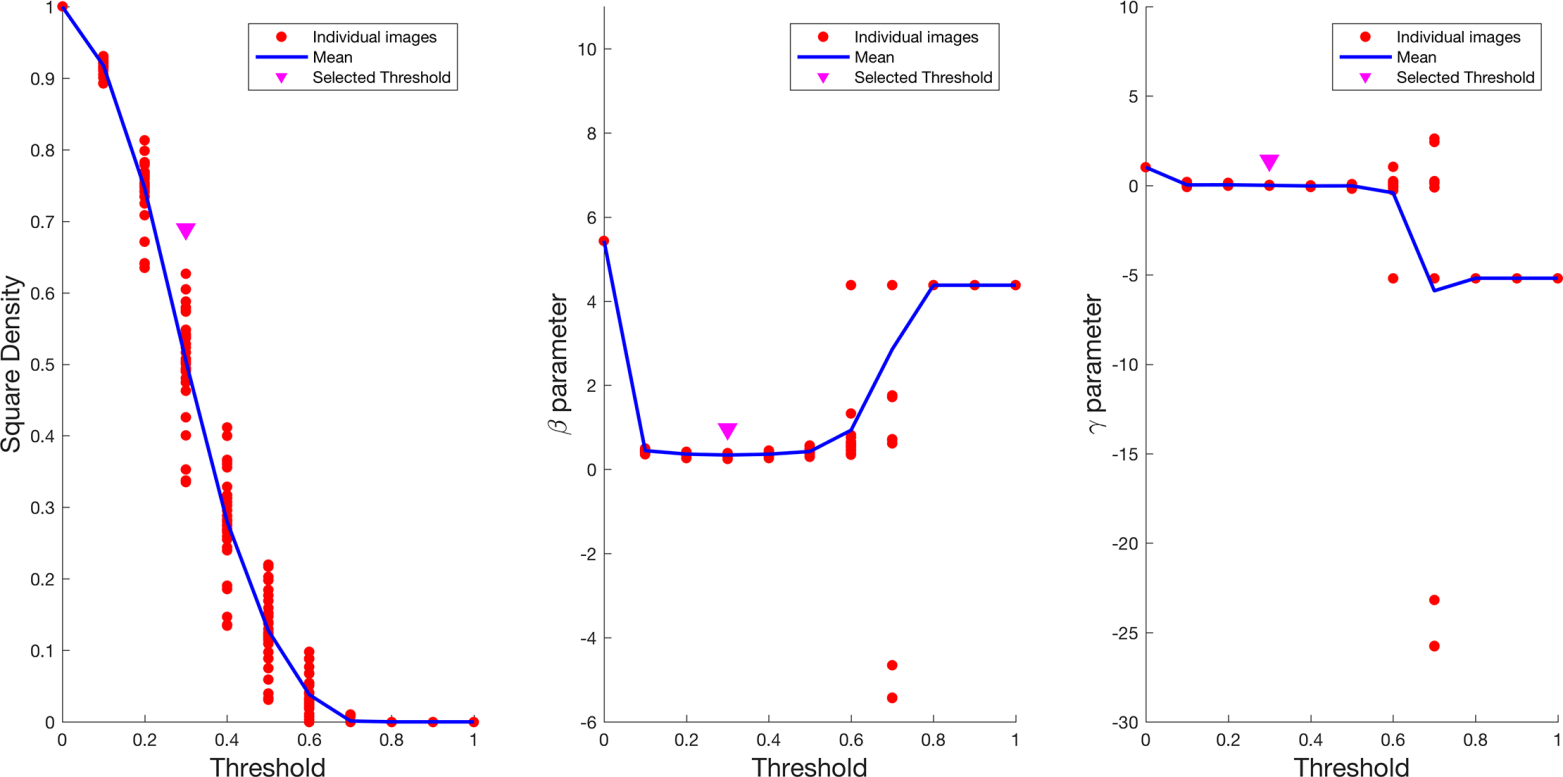
Effect of threshold selection on Ising model parameters and square density. The figure shows how parameter estimates from the Ising model change when using different threshold values to obtain binary images from decorrelation agiograms of the choriocapillaris. The threshold has been increased at 0.1 steps and applied to all images each time to calculate the density. In each plot, the blue line represents the mean of the parameter estimates at each threshold value, the red dots represent the value calculated for each image and the pink arrow point highlights the threshold value used in this study. The plot on the far left shows how the density estimate on the square area used for the Ising model calculations changes with different threshold values. As for the plot in Fig 3 the selected threshold is centered on the sigmoid shaped curve of the mean density values. The middle and rightmost plots show how parameter estimates from the Ising model change when using different threshold values. The selected threshold is in the center of the interval where stable measures are obtained. Note how the parameter estimates become unstable with extreme threshold values.

As for the Standard Density, to explore the effects of threshold selection on the Ising parameter estimates we calculated parameter values while changing the threshold used to binarize the choriocapillaris image. Density value on the square region (denoted as Square Density) used to calculate the Ising parameters (see Methods) was also calculated. Results are depicted in Fig 6. Ising parameters were very unstable at extreme threshold values (below 0.1 and above 0.5). Similarly to the Standard Density, the mean Square Density value decreased at increasing threshold values in a sigmoid fashion. Fig 6 shows how the selected threshold (0.3) lies on the steepest part of the sigmoid curve and in the center of the rage where stable parameter estimates for the Ising model could be obtained.

**Fig 7:**
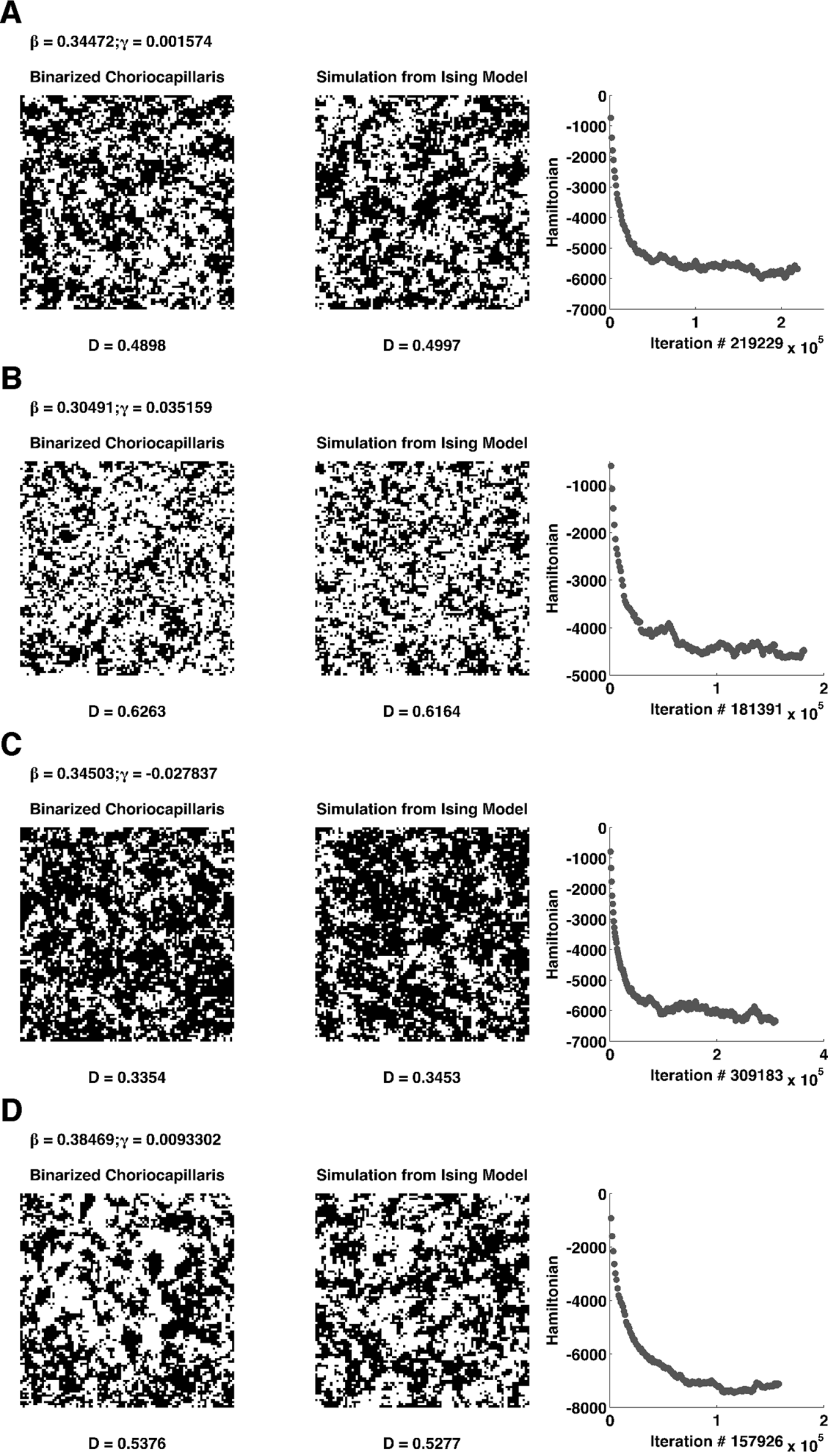
Simulation from the Ising model of different choriocapillaris textures. Fig 7.A-D shows simulation from the Ising model using a Metropolis–Hastings sampler. On the left, the original image from a healthy subject, on the center the simulation from the Ising model trained on the original texture. On the far right, a graph showing how the Hamiltonian of the system decreases as the simulation goes on (sampled every 1000 iterations). Estimated coefficients for the Ising model are reported on the top of each original image, the white pixel density (denoted as D) is reported below each respective choriocapillaris image, both original and simulated.

We then used simulations from the MH-MCMC (see Methods) to compare the results from the estimated model with the real textures. Fig 7 provides some representative results from the simulation (on the left) compared with the original binary image (in the center) the parameters were estimated from. On the right side, the graphs show the Hamiltonian of the system decreasing as the simulation proceeds. One crucial point was to define a stopping criterion for the simulation: indeed, there is a rapid decay of the Hamiltonian during the first 100000 iterations, but, upon several trials, a stable Hamiltonian did not guarantee that the simulation had reached the target density. One good criterion that we have adopted is to wait 150000 iterations and then stop the simulation whenever it reached a density close to the original image (with a tolerance of ± 0.01). This procedure ensured that the spatial properties were accurately represented before stopping the MCMC based on the density alone. In some cases, with extreme values for the parameters the MCMC took a long time to converge to the original density (more than 300000 iterations).

## Discussion

In our work, we performed an in depth analysis of the features of the choriocapillaris in normal eyes as visualized with OCT-A. We primarily focused on a correct estimation of the density of the subfoveal choriocapillaris. Although measurements of the choriocapillaris density (with a method comparable to ours) have already been provided [5], the choice of the ROI circular area to perform calculations has been somehow arbitrary. Here, for the first time, we precisely analyze how the choice of the ROI area affects the precision of the measurements. By selecting a ROI radius of 450 μm (45 pixels) we achieved a strong reduction in the measurement variability while avoiding the extrafoveal choriocapillaris to influence the measured density. This is of particular importance, since the extrafoveal choriocapillaris is acknowledged to have different features, both in terms of density and appearance [16], while carrying a significant risk of including projection artifacts from superficial thicker vessels. Defining a standard for density measures is an important objective, given the increasing importance of OCT-A technology in investigating the living eye vasculature both in normal and diseased eyes [16–18], in order to obtain comparable results both for clinical and scientific purposes. Compared to Wang et al (density within the 1 mm ring 0.45 ± 0.03), our results show higher density values and higher intersubject variability [5]. Since Wang et al. used a similar area size for the measuring ROI (1 mm diameter) compared to ours (0.9 mm) it is difficult to identify an obvious reason for this discrepancy: possible explanations could rely in the different population analyzed, the different sample size (much bigger in Wang et al.) and slight differences in the measuring technique (most importantly threshold settings). In this perspective, we also provided a detailed analysis of how threshold selection can affect our measurements. We chose a fixed threshold on equalized images rather then an adaptive threshold, so we could easily change the threshold value and analyze the effect on calculated density values. The chosen threshold (0.3) resulted to be located in the steepest part of the sigmoid curve, as depicted in Fig 3. This is important, since the steepest point on a sigmoid curve is the one that theoretically allows the largest variability when measuring density in different subjects and/or eyes. Allowing larger potential intersubject variability might yield a better discriminative power between healthy and unhealthy subjects, but these hypothesis needs to be confirmed with analyses on large cohorts of patients with specific diseases.

Standard Density was significantly (although weakly) negatively correlated with the age of the subject (p < 0.05) but not with the SSI, possibly indicating that the measure is able to capture the normal aging process of the choriocapillaris. This analysis should be interpreted carefully, since a significant (and stronger) correlation existed between age and SSI. In this sense, a correlation between density and SSI is expected, even in the case of no effect of the SSI on the measure itself, simply due to an indirect effect of aging. To determine the impact of signal strength on the measurements an analysis on a group of subjects with comparable ages and different degrees of media opacity (i.e. cataract) would be needed.

Since there has been no attempt, to our knowledge, to give a quantitative description of the structural characteristics of the choriocapillaris, we tried and develop a model that could capture the essential structural behavior of this vascular layer. MRF models have been widely used in image processing[9, 19, 20] both for modeling and segmentation. When it comes to binary images, the Ising model is a natural choice as it is a very flexible model able to represent a great variety of textures. This is clearly shown in Fig 5, where the images of the binarized choriocapillaris differ greatly in terms of clustering and density and are all adequately synthetized by the Ising model. Having a flexible model allowed a full characterization of complex images with only two parameters. This is not only important for description, but can also be used for constructing normative databases of the parameters for evaluating choriocapillaris images for diagnostic purposes or as a generating model to obtain random realization of choriocapillaris maps with desired characteristic for theoretical perfusion analysis. Indeed, density and clustering of the texture are easily controlled by modifying the *β* and *γ* parameters, the second being strongly correlated with the textural density (Fig 5). It is difficult to definitely explain the mutual correlation between the *β* and *γ* parameters. One possible explanation might rely in the fact that the clustering and the density parameters operate in concert in determining the final density, i.e. higher clustering requires only minimal contribution from the density parameter; with high clustering, however, the *γ* parameters might have dramatic effects since small changes might determine the switch of an unstable system from the high density to the low density configuration, significantly affecting the results of the texture synthesis process. Such behaviors might be better outlined by an extended simulation analysis. Indeed, in this perspective, the relationship between the two parameters could be much more effective in identifying pathological conditions with extended choriocapillaris atrophy rather than the values of the single parameters alone.

One limitation of this method is the difficulty in deciding a stopping criterion for the texture generation when the target density is not known, since there is no way to decide if the algorithm has converged. One possible solution relies in the high predictive power of the estimated *γ* parameter for the original density (Table 2 and Fig 5B), effectively allowing an estimation of target density that can be used as a stopping criterion. A second limitation might be the inclusion of projection artifacts in the density measures and structural analyses of the choriocapillaris. However, as in our data, this could be avoided by restricting the area of analysis to the subfoveal region that is not overtopped by major superficial vessels, together with the exploitation of artifact suppression algorithms provided by many visualization softwares [21] or more advanced artifact suppression strategies [22]. These precautions were sufficient in our dataset to avoid the influence of any evident projection artifacts from superficial layers. It has to be noted, however, that, even if the whole choriocapillaris image could be safely analyzed, the current implementation of the model might not be adequate to describe the behavior of other parts of the choriocapillaris outside the foveal region. Location dependent variation of the parameters of the model might be necessary to account for structural changes in the peripheral choriocapillaris.

Future developments will be to increase the sample size of normal subjects to have a consistent database for the normal density and texture parameters and to develop a continuous valued spatial model for the choriocapillaris.

We strongly believe that such an approach is able to offer clinicians a compact, yet exhaustive, description of this very complex vascular layer. Recently, Spaide showed that the choroidal flow imaged with OCT angiography can develop perfusion voids over time due to aging [23], but a dynamical model describing this phenomenon is still lacking. The Ising model could provide an effective method to model the aging process over time on follow up series of choriocapillaris images. Indeed, the γ parameter showed a good correlation with the overall density and could be used in a time dependent model to account for normal density reduction in the aging choriocapillaris and detect (or possibly predict) deviations toward pathological modification of the choriocapillaris, while the β parameter might account for abnormal clustering of dark pixels, identifying pathological perfusion void development or account for different choriocapillaris structures, as in myopic eyes [24].

Further investigations will be needed to understand how the density measure and the Ising parameters can change in different retinal diseases. Microvascular alterations of choriocapillaris have been reported, for example, in central serous retinopathy [25] and in geographic atrophy [26]. Although in those cases pathological changes were readily detectable (especially in geographic atrophy) texture analysis might help in detecting small precocious modifications that might provide fast detection of patients at risk and a better understanding of the pathophysiological mechanisms underlying the degeneration process, especially in terms of perfusion impairment, that would be otherwise not detected.

## Supporting information

S1 Data Repository Data used for the analysis. Readable table containing all the density estimates used in this study, the results of the Ising model, the signal strength index (SSI) for each image, the age of each subject and the size of the foveal avascular zone (FAZ).
(CSV)
